# Oligomerization and insertion of antimicrobial peptide TP4 on bacterial membrane and membrane-mimicking surfactant sarkosyl

**DOI:** 10.1371/journal.pone.0216946

**Published:** 2019-05-13

**Authors:** Shih-Han Wang, Chiu-Feng Wang, Ting-Wei Chang, Yu-June Wang, You-Di Liao

**Affiliations:** Institute of Biomedical Sciences, Academia Sinica, Taipei, Taiwan; Nanyang Technological University, SINGAPORE

## Abstract

Antimicrobial peptides (AMPs) are important components of the host innate defense mechanism against invading microorganisms. Although AMPs are known to act on bacterial membranes and increase membrane permeability, the action mechanism of most AMPs still remains unclear. In this report, we found that the TP4 peptides from Nile tilapia anchored on *E*. *coli* cells and enabled them permeable to SYTOX Green in few minutes after TP4 addition. TP4 peptides existed in small dots either on live or glutaraldehyde-fixed cells. TP4 peptides were driven into oligomers either in soluble or insoluble form by a membrane-mimicking anionic surfactant, sarkosyl, depending on the concentrations employed. The binding forces among TP4 components were mediated through hydrophobic interaction. The soluble oligomers were negatively charged on surface, while the insoluble oligomers could be fused with each other or piled on existing particles to form larger particles with diameters 0.1 to 20 μm by hydrophobic interactions. Interestingly, the morphology and solubility of TP4 particles changed with the concentration of exogenous sarkosyl or trifluoroethanol. The TP4 peptides were assembled into oligomers on or in bacterial membrane. This study provides direct evidence and a model for the oligomerization and insertion of AMPs into bacterial membrane before entering into cytosol.

## Introduction

Most conventional antimicrobials inhibit the synthesis of bacterial nucleic acid, protein or cell wall components. However, the widespread use of antibiotics in both medicine and agriculture has contributed to the emergence of drug-resistant bacteria [1, 2]. Thus, development of new antimicrobials with unique targets and action mechanisms different from those of conventional antibiotics is an acute and urgent need. Natural antimicrobial peptides/proteins (AMPs) with the ability to disrupt membrane integrity have been isolated from multiple sources including bacteria, fungi, insects, invertebrates and vertebrates [2–4]. Some bioactive peptides do not merely act as direct antimicrobial agents but also represent important effectors and regulators of the innate immune system with similarity to LL-37 [5]. Until now, several AMPs have been under phase II/III trials for the prevention and treatment of microbial infections [6–8].

Thousands of AMPs have been isolated and investigated for several decades with emphasis on the structure of AMPs and the membrane integrity of bacteria. Despite the fact that secondary structures and amino acid sequences of AMPs are diverse, they usually possess amphipathic structure with hydrophobic and cationic face at each side of the AMP within hydrophobic environments [9–11]. The cationic residues are believed to bind anionic components of the bacterial membrane and cell wall through electrostatic interactions, while the hydrophobic residues bind to the lipid face of membranes by hydrophobic forces. Various targets of AMPs have been extensively studied such as outer membrane proteins, lipids, the inner membrane, inner membrane proteins, nucleic acids as well as intracellular proteins [7, 12–14]. With respect to the disruption of membrane integrity, several models have been proposed such as the barrel-stave model, toroidal model, carpet model, and detergent-like model based on the interactions between AMPs and artificial membranes at hydrophobic environments [15–17]. Many AMPs exist as non-structural forms in aqueous solution and adapt into amphipathic structures in membrane-mimicking environments such as surfactants [17].

Our previous results showed that the anionic surfactant sarkosyl at 2.6 mM was able to drive the synthetic AMP GW-Q6 and natural cationic AMP TP4 from Nile tilapia into amphipathic structures having an α-helix [18–20]. Both AMPs were able to bind receptor proteins on the outer membranes of Gram-negative bacteria at 2.6 mM such as OprI from *Pseudomonas aeruginosa* and Lpp from *E*. *coli* [19–23]. Both GW-Q6 and TP4 peptides exhibit helical structures as determined by CD spectrum analyses and NMR studies [19, 20]. Here, we further show that TP4 peptides are driven into oligomers and even vesicles by the membrane-mimic surfactant sarkosyl when inserted into the bacterial membrane in the form of oligomers.

## Materials and methods

The following oligopeptides, TP4 (FIHHIIGGLFSAGKAIHRLIRRRRR), GW-Q6 (GIKIAKKAITIAKKIAKIYW), FITC-conjugated TP4, Rhodamine-conjugated TP4 and biotinylated GW-Q6 at N-termini, were synthesized by Kelowna International Scientific Inc. (Taipei, Taiwan) with more than 95% purity and their molecular masses were verified by mass spectrum analysis. Streptavidin gel was purchased from GE Healthcare (Uppsala, Sweden). Sodium N-dodecanoylsarcosinate (sarkosyl) was supplied by Wako Pure Chem. (Osaka, Japan). Glutaraldehyde and paraformaldehyde were purchased from Sigma-Aldrich Inc. Diethylaminoethyl-cellulose (DE52) resin and carboxylmethyl-cellulose (CM52) resin were purchased from Whatman International Ltd. (Maidstone, England). 2,2,2-trifluoroethanol (TFE) was purchased from Acros Organics (New Jersey, USA). Uranyl acetate was obtained from EMS (Pennsylvania, USA).

### Antimicrobial activity assays

*E*. *coli* K-12 (MG1655) was grown overnight in Luria-Bertani broth, washed and diluted to 5x10^6^ colony forming units (cfu)/ml in phosphate-buffered saline (PBS). Then serially diluted TP4 or FITC-TP4 peptides (5 μl) were mixed with the microbes (2.5x10^5^ cfu/45 μl) and incubated at 37°C for 1.5 hr. Serial dilutions of each AMP-treated bacteria were plated on agar plates for the determination of remaining cfu [14, 24]. At least three independent experiments were performed for each assay to determine the average value with standard error.

### Assays for permeability and membrane binding

For permeability assay, overnight-cultured *E*. *coli* was prefixed by 0.2% glutaraldehyde (GA), washed and suspended in distilled water (1x10^7^ cfu/ml). Both GA-fixed and non-fixed *E*. *coli* cells (100 μl in a 96-well microplate) were incubated with 1 μM SYTOX Green (Molecular Probes) for 5 min in the dark before TP4 addition. The fluorescence of SYTOX Green bound to cytosolic DNA was determined by a SpectraMax M2 microplate reader (Molecular Devices, CA, USA) with excitation at 485 nm and emission at 520 nm (21). For bacterial binding assay, aliquots of FITC-TP4 (0.5 μg, Mr = 3484 Da) and FITC (0.1 μg, Mr = 389 Da) were incubated with non-fixed or fixed *E*. *coli* cells (1x10^7^ cfu/200 μl) with fluorescence in a high precision cuvette (Hellma Analytics, Mulheim, Germany) and were measured by an FP-8500 fluorescence spectrophotometer (Jasco, Tokyo, Japan) with excitation at 480 nm and emission at 520 nm. For the TP4-binding capacity of bacteria, GA-fixed and non-fixed *E*. *coli* (13x10^7^ cfu/200 μl) were incubated with increasing amounts of FITC-TP4 as indicated for 10 min. The fluorescence of FITC-TP4 bound to bacteria after centrifugation (300×*g*, 5 min) was suspended and measured by FP-8500 with excitation at 480 nm and emission from 505 to 600 nm. To see the effects of bacterial concentration on the absorption of AMPs in solution, increasing amounts of non-fixed and fixed *E*. *coli* cells as indicated were added to FITC-TP4 solution (2 μg/200 μl), the fluorescence of FITC-TP4 in the supernatant and bacteria after pulled down and resuspension were measured as mentioned above.

### Confocal laser-scanning microscopy (CLSM)

#### FITC-TP4 particles in *E*. *coli*

For observation of FITC-TP4 peptide in *E*. *coli* K-12, the bacteria (1x10^7^ cfu) were inoculated on a chamber slide in 100 μl phosphate buffer (10 mM sodium phosphate, pH 7.4) at room temperature for 2 hr, then prefixed with 0.2% glutaraldehyde. FITC-TP4 (5 μg) was added to the pre-fixed or non-fixed bacteria for 10 min and further fixed with 4% paraformaldehyde/2.5% glutaraldehyde before examination under confocal microscope CLSM 700 (ZEISS, Oberkochen, Germany).

#### Morphology of TP4 particle in sarkosyl buffer

For observation of FITC-TP4 peptide in various sarkosyl buffers, 4 μg of TP4 peptides was dissolved in 20 μl sarkosyl buffer (0.25x, 0.5x and 1x Sar) and placed on glass slide for 30 min before examination. 1x Sar buffer contains 10 mM sodium phosphate, 100 mM NaCl, pH 7.4 and 2.6 mM sarkosyl (0.075% sarkosyl, w/v). Similarly, various concentrations of FITC-TP4 peptides (1, 4 and 16 μg/20 μl) or sarkosyl as indicated were also employed for the study. The morphologies and images of FITC-TP4 or Rhodamine-TP4 particles under various environments were observed and taken by CLSM 780 (ZEISS, Oberkochen, Germany).

#### Cross sections of TP4 particle

The FITC-TP4 particles (6 μg in 20 μl 0.5x Sar) with diameter larger than 10 μm on cover slide were selected for study. Cross sectional images of the TP4 particle were taken along the Z-axis with intervals of 0.1 μm by fluorescence microscope (CLSM 780). Three-dimensional model of TP4 particle was obtained from these sectioned images using ZEN (blue edition) microscope software (ZEISS, Germany). The internal structures of the TP4 particle was also shown by the cut-off plane along the X-axis.

#### Morphological changes of FITC-TP4 particles under various environments

To observe changes in the FITC-TP4 particle, 4 μg were dissolved in 20 μl 0.5x Sar and loaded on round glass slide (3 cm in diameter) for 30 min to allow for vesicle formation. Equal volumes (20 μl) of 1.5x Sar buffer was added to the margin of TP4 drop (0.5x Sar) to make 1x Sar, in which TP4 particles were disrupted. In contrast, FITC-TP4 peptides (4 μg) were dissolved in 20 μl 1x Sar and loaded on glass slide, then diluted with equal volumes of 0x Sar buffer for the formation of TP4 particles. Similar to previous experiments, the sarkosyl solution was drained from the glass slide (3 cm in diameter) once the FITC-TP4 particles had been adhered on it and replaced by 20 and 30% TFE. The images of TP4 particle under various environments were taken by CLSM780 at different time intervals.

### Bacteria binding assay

TP4 peptides (8 μg) were incubated with 100 μl (5x10^7^ cfu) non-fixed and 0.2% GA-fixed *E*. *coli* in 10 mM sodium phosphate for 10 min at room temperature. The TP4-treated bacteria after washing by 10 mM sodium phosphate were incubated with 1x Sar, 4x Sar, or various NaCl concentrations for 10 min. The retained TP4 peptides on bacteria were dissolved and analyzed by SDS-PAGE and Coomassie Blue staining.

### Protein identification

The proteins extracted from glutaraldehyde-fixed *E*. *coli* were analyzed by 15% SDS-PAGE. The protein bands of interest were excised and cut into small pieces (1x1 mm). The gel pieces were dehydrated, reduced and alkylated before trypsin digestion. The trypsin-digested peptides were subjected to LC-MS/MS analysis by an anoACQUITY UPLC System (Waters, USA) coupled to a high-resolution mass spectrometer (Orbitrap Elite, Thermo Fisher Scientific, USA). The MS/MS spectra were searched with the Mascot engine (v2.6, Matrix Science, UK) against the UniProtKB Escherichia coli protein database [25].

### Crosslinking of TP4 peptides

TP4 peptides (2 μg) were dissolved in 10 μl sarkosyl solution, incubated with 0.1% glutaraldehyde at 37°C for 30 min and subjected to SDS-PAGE followed by Coomassie Blue staining. To explore the stability of TP4 assembly, NaCl or TFE was added to the 1x Sar solution at the concentration as indicated before crosslinking with GA. For the crosslinking of TP4 peptides on or in *E*.*coli* K-12 (MG1655), the bacteria were pre-fixed with 0.2% glutaraldehyde in 100 μl for 50 min at room temperature, then incubated with 40 μg TP4 peptide for 10 min and further crosslinked with 0.1% glutaraldehyde. The bacteria were extracted by 4x Sar (0.3%, w/v) and centrifuged at 3,300 ×*g* for 5 min. The TP4 peptides as well as bacterial proteins in the supernatant and pellet were subjected to SDS-PAGE and Coomassie Blue staining.

### Binding of AMPs by biotinylated AMPs

Streptavidin-conjugated beads were incubated with 6 μg of biotinylated GW-Q6 peptides in 600 μl 1x Sar buffer (10 mM sodium phosphate, 100 mM NaCl and 0.075% sarkosyl) for 3 hr on a rolling wheel. The immobilized biotinylated AMPs were further mixed with TP4 peptides (4 μg) in a buffer as indicated at 4°C overnight on a rolling wheel, washed twice with buffer and subjected to SDS-PAGE and Coomassie Blue staining (22). To determine the stability of AMP complexes, various concentrations of NaCl or TFE were included in the washing buffer.

### Pull down of TP4 peptides by DE52/CM52 resins

TP4 peptides (10 μg) dissolved in 600 μl sarkosyl buffers (0x, 0.25x, 0.5x and 1x Sar) were centrifuged at 13,200 ×*g* for 5 min. The supernatants were further incubated with DE52 or CM52 resins in sarkosyl solution at room temperature for 1 hr on a rolling wheel. The TP4 peptides bound to the resins after washing twice and the previously mentioned pellets were analyzed by SDS-PAGE and Coomassie blue staining. To determine the stability of TP4 peptides bound to resin, various concentrations of TFE (0, 10, 20 and 30%, v/v) were included in washing buffer.

### Morphology of TP4 particles when analyzed by transmission electron microscopy (TEM)

For observation of TP4 peptide in sarkosyl solution, 4 μg of TP4 peptides was dissolved in 20 μl sarkosyl buffer (0.25x, 0.5x and 1x Sar), fixed by 0.2% glutaraldehyde, then loaded on Nickel grid (75 mesh) using a filter paper to blot off excess solution. The TP4 peptides on the grid were air dried, stained with 2% uranyl acetate and examined under a transmission electron microscope JEM-1200EX (JEOL, Tokyo, Japan).

### Stability of TP4 vesicles in various TFE concentrations

To explore the stability of TP4 particles, TP4 peptides (8 μg) were dissolved in 75 μl 0.5x Sar and loaded in polypropylene (PP)-based Eppendorf tube for 30 min at room temperature, then centrifuged at 13,200 ×*g* for 5 min. Various concentrations of 100 μl TFE (0, 10, 20, 30, 40, 50%) were employed to resuspend the pellet and incubated at room temperature for 1 hr with gentle shaking. The supernatant collected after centrifugation at 13,200 ×*g* for 5min was transfered to a new Eppendorf tube for vacuum drying. Both the vacuum-dried sample and TP4 peptides remained in the original tube were analyzed by SDS-PAGE with Coomassie Blue staining.

## Results

### Attachment of TP4 peptides on bacteria

**Fig 1:**
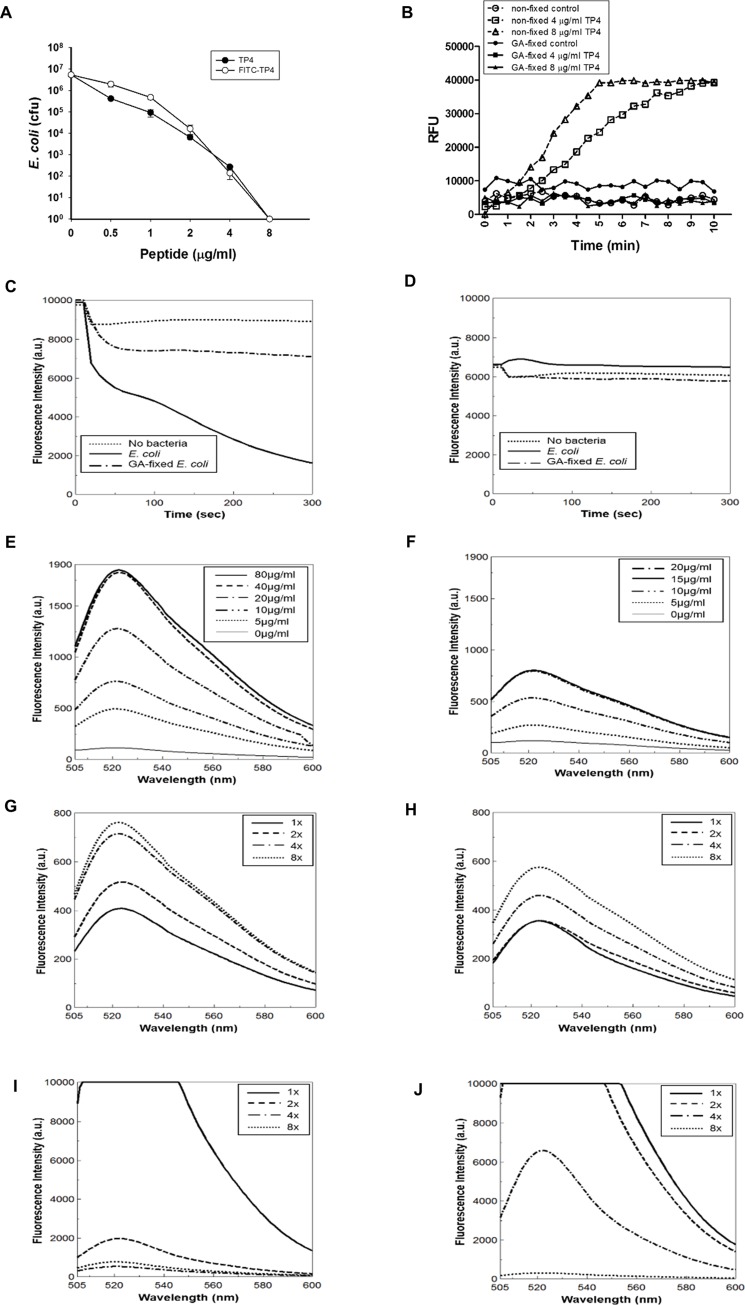
Binding and entry of TP4 peptides in *E*. *coli*. (A) TP4 and FITC-TP4 peptides exerted similar bactericidal ability against *E*. *coli*. 50 μl of *E*. *coli* (5x10^6^ cfu/ml) was incubated with serial diluted TP4 and FITC-TP4 peptides for 1.5 hr, and the remaining viable cells were determined after plating on agar plate. (B) Live *E*. *coli* cells became permeable after TP4 treatment. The non-fixed *E*. *coli* (1x10^7^ cfu/ml) incubated with TP4 (4 and 8 μg/ml) in 200 μl were permeable to SYTOX Green, while the glutaraldehyde (GA)-fixed *E*. *coli* cells were not. (C, D) Time course depletion of FITC-TP4 and FITC from solution by *E*. *coli* cells. FITC-TP4 (2.5 μg/ml) and FITC (0.5 μg/ml) were incubated with non-fixed (1x10^7^ cfu) and GA-fixed *E*. *coli* cells in 200 μl. The fluorescence of FITC-TP4 (C) was dramatically decreased by non-fixed *E*. *coli* rather than GA-fixed *E*. *coli*, while the fluorescence of FITC (D) was not affected by both cells. (E, F) FITC-TP4 binding capacity of *E*. *coli* cells. The GA-fixed (F) and non-fixed *E*. *coli* (E) (13x10^7^ cfu in 200 μl) were incubated with increasing amount of FITC-TP4 as indicated for 10 min. The binding capacity of non-fixed bacteria (40 μg/ml) was higher than that of fixed bacteria (15 μg/ml). (G-J) The movement of FITC-TP4 from solution to bacteria. FITC-TP4 (2 μg/200 μl) was incubated with increasing amounts of non-fixed *E*. *coli* (G, I) and fixed *E*. *coli* (H, J) as indicated. The fluorescence of FITC-TP4 bound to bacteria (G, H) and left in solution (I, J) were measured after centrifugal resuspension. The concentration of 1x bacteria is 8x10^7^ cfu/ml *E*. *coli*.

To see the behaviors of antimicrobial peptide TP4 on bacterial membrane and inside the bacterium, an N-terminal-labelled fluorescence peptide, FITC-TP4, as well as non-labelled TP4 peptide was employed for this study. Both peptides exerted similar bactericidal activity against *E*. *coli* (Fig 1A). The live bacteria became permeable to a DNA-binding dye, SYTOX Green, about 3–5 min after TP4 addition, however, the glutaraldehyde (GA)-fixed cells (0.2%, 20 mM) were not permeable to the dye (Fig 1B). The free FITC-TP4 peptides in solution were dramatically reduced in 2~5 min after the addition of live bacteria, while most of them remained in solution (~80%) if GA-fixed cells were added (Fig 1C). With respect to FITC dye only, no significant changes were observed regardless of the addition of live or GA-fixed cells (Fig 1D). The amount of FITC-TP4 bound to live cells or GA-fixed cells increased with the concentration of peptides, and the maximal binding capacity of live cells was about two-folds higher than that of GA-fixed cells in the presence of excess amounts of FITC-TP4 (Fig 1E and 1F). Similarly, the amount of FITC-TP4 bound to live or GA-fixed bacteria also increased with the concentration of bacteria, and that live cells were also higher than that to GA-fixed cells (Fig 1G and 1H). Interestingly, the amount of FITC-TP4 remaining in solution decreased with the increase of exogenous bacteria, and that live cells left in solution were less that of GA-fixed cells (Fig 1I and 1J).

**Fig 2:**
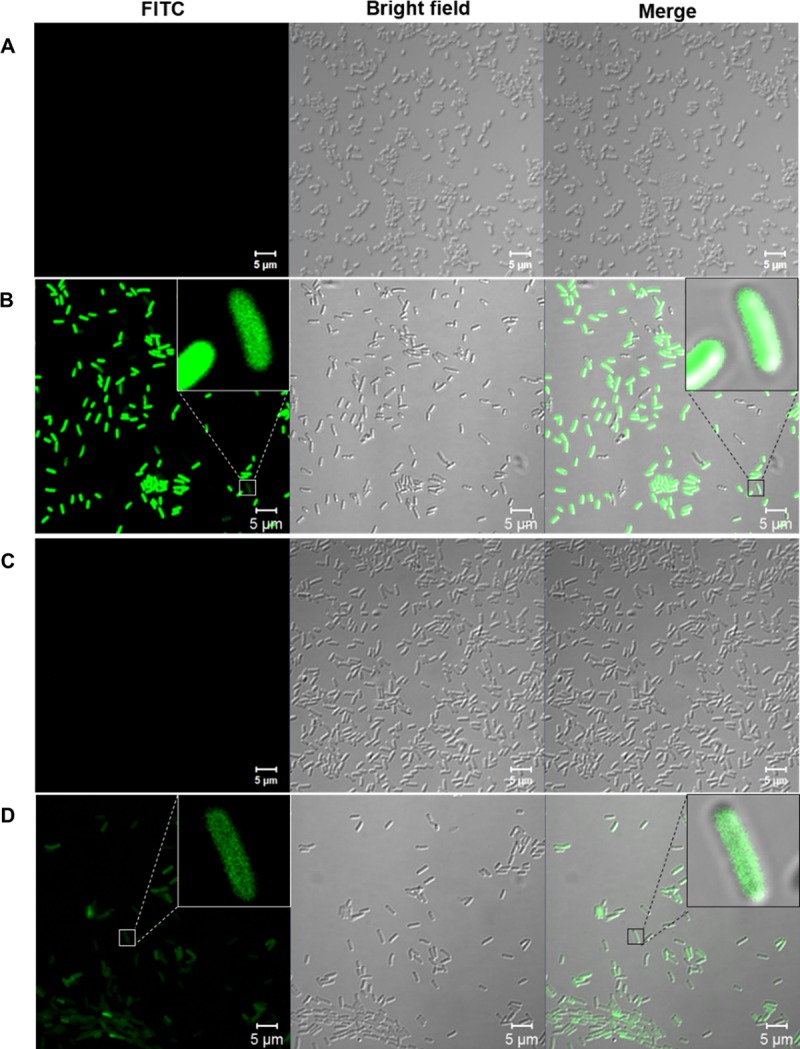
TP4 peptides existed in small dots in *E*. *coli* under fluorescent confocal microscope. The non-fixed (B) and GA-fixed (D) *E*. *coli* (1x10^7^ cfu) were incubated with 5 μg FITC-TP4 peptides in 200 μl phosphate buffer and examined under confocal fluorescence microscopy CLSM 700. No peptide was added in non-fixed (A) and glutaraldehyde-fixed (C) *E*. *coli* cells. Small dots are shown in the insets of panel B and D at high magnifications.

The FITC-TP4 peptides were apparently internalized into the cytosol 10 minutes after peptide treatment when examined under confocal fluorescent microscope. However, the efficiency of peptide entry was markedly reduced if the bacteria were prefixed with 0.2% GA (20 mM) before peptide addition. Interestingly, the FITC-TP4 peptides existed in small dots which can be seen in the lateral region of GA-fixed bacteria (high magnificent inset of Fig 2D) as well as in the whole live bacteria (Fig 2B). For control experiments, no signals were observed in both non-fixed and GA-fixed bacteria without peptide addition (Fig 2A and 2C). These results indicate that FITC-TP4 peptides are assembled into clusters on bacterial membrane and also in the cytosol of live cells because individual FITC-TP4 molecules are not visible under fluorescent microscope.

### Insertion of TP4 peptides into bacterial membrane

**Fig 3:**
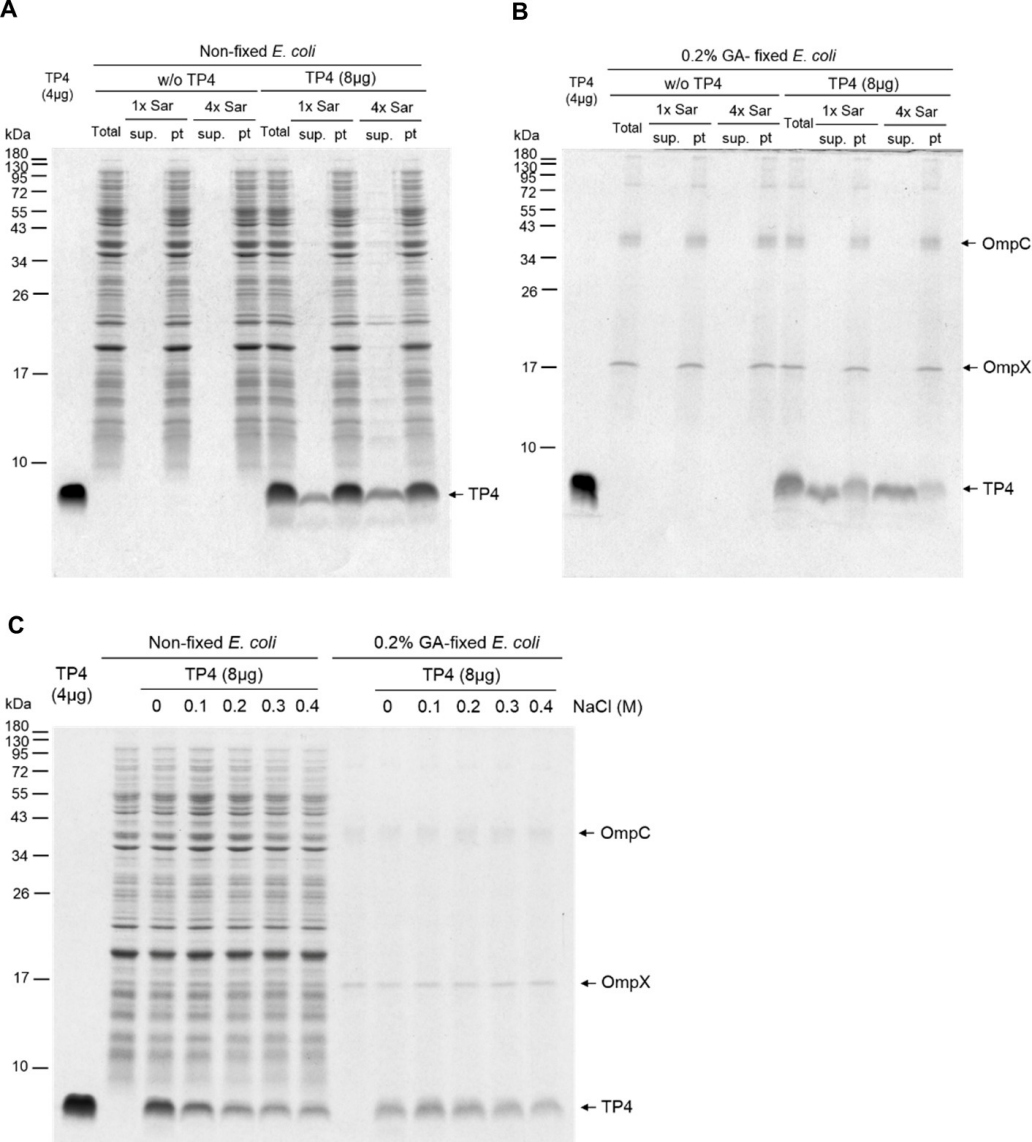
Binding of TP4 peptides to *E*. *coli* membrane and its susceptibility to surfactant. (A, B) Binding of TP4 peptides to *E*. *coli* and susceptibility to Sar extraction. The GA-fixed (B) and non-fixed (A) *E*. *coli* (8x10^7^ cfu/100 μl) were treated with TP4 peptides (8 μg) for 10 min and extracted with 1x or 4x Sar for 10 min. The compositions of the extract (sup.) and insoluble pellet (pt) were analyzed by SDS-PAGE and Coomassie blue staining. Two outer membrane proteins, OmpC and OmpX, of *E*. *coli* could be extracted from GA-fixed bacteria by SDS-loading buffer but not by 1x or 4x Sar. (C) Susceptibility of TP4 binding to NaCl elution. The binding of TP4 peptides to non-fixed *E*. *coli* were susceptible to the elution by NaCl while that to GA-fixed *E*. *coli* was resistant to salt elution. Total, sup. and pt represent total lysate, supernatant and pellet, respectively.

To determine the location of TP4 peptides in the bacteria, the TP4 peptides bound to non-fixed and GA-fixed *E*. *coli* cells were extracted by a mild anionic surfactant, sarkosyl, which was unable to solubilize bacterial proteins from either GA-fixed or non-fixed *E*. *coli* cells. Only small portions of bound TP4 peptides were extracted by 1x Sar (0.075%, w/v) or 4x Sar (0.3%, w/v) solution from non-fixed bacteria, while the majority of bound TP4 peptides were still retained on the bacteria (Fig 3A). Interestingly, more than half of the bound TP4 peptides on GA-fixed bacteria were extracted by 1x Sar solution and the majority of them was extracted by 4x Sar solution (Fig 3B). It is of note that two outer membrane proteins, OmpX and OmpC as identified by mass spectrum analysis, remained in GA-fixed *E*. *coli* after Sar solution could be extracted by hot SDS-PAGE loading buffer containing 2% SDS (w/v) (Fig 3B and S1 Table). To further investigate the binding forces of TP4 to bacteria, we find that more than half of bound TP4 peptides on non-fixed cells were removed by 0.2–0.4 M NaCl, while those on GA-fixed bacteria was resistant to NaCl elution (Fig 3C). These results suggest that the binding of TP4 peptides to the membrane of GA-fixed bacteria are mainly mediated through hydrophobic interaction, while the charged molecules on membranes of live bacteria are mediated by both electrostatic and hydrophobic interactions.

### *In vitro* oligomerization of TP4 in the presence of sarkosyl

**Fig 4:**
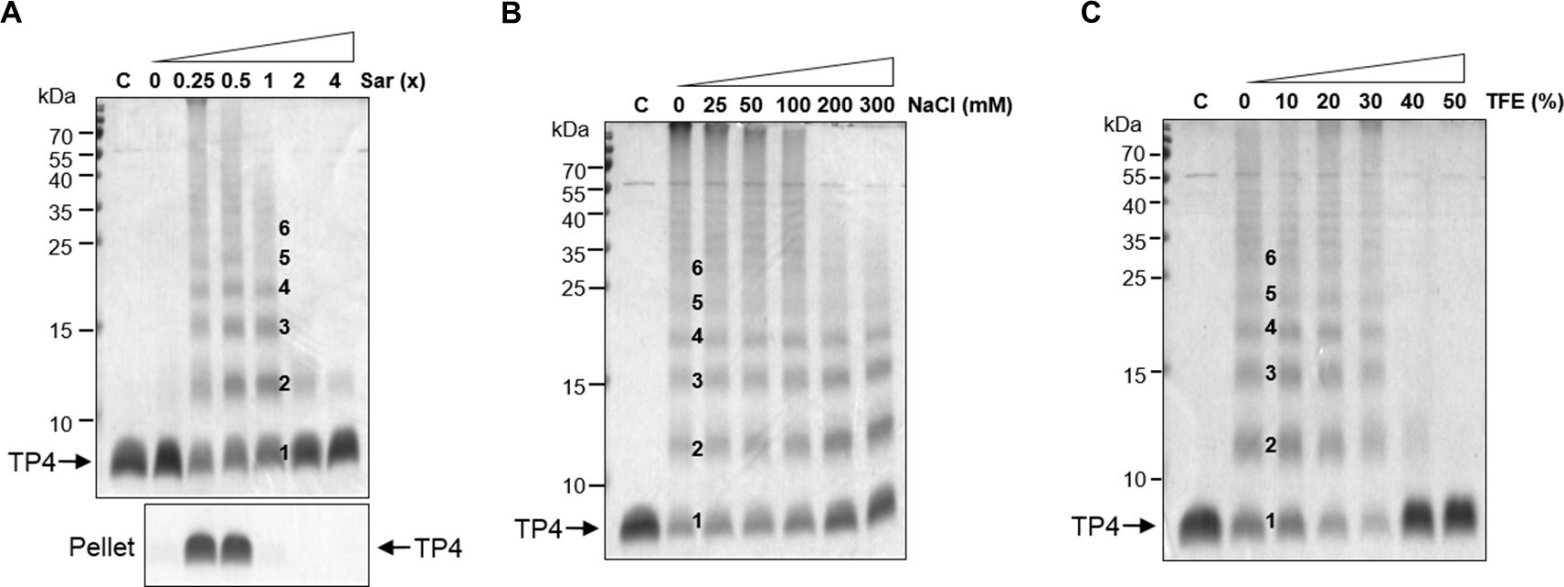
Oligomerization of TP4 peptides in the presence of sarkosyl. (A) Oligomerization of TP4 peptides at various sarkosyl concentrations. TP4 peptides (4 μg) were dissolved in 10 μl of 0, 0.25x, 0.5x, 1x, 2x and 4x Sar, crosslinked with 0.1% glutaraldehyde for 30 min and subjected to SDS-PAGE. TP4 became oligomers from monomers, dimers to hexamers as shown by 1–6 although they precipitated at 0.25x and 0.5x Sar. Pellets represent the precipitates of 4 μg TP4 peptides in 200 μl sarkosyl solutions without crosslinking. (B, C) Stability of TP4 oligomers to NaCl (B) and TFE (C). TP4 peptides were dissolved in 1x Sar containing various concentrations of NaCl and TFE as indicated for 30 min before crosslinking.

To investigate the status of TP4 peptides on bacterial membrane, an anionic and amphipathic surfactant, sarkosyl, was employed to mimic the membrane environment. Here we find that TP4 peptides were soluble and clear in appearance at 1x Sar, or without sarkosyl (0x sar), while they were opaque and could be spun down at lower concentrations of sarkosyl (0.5x, 0.25x Sar) (Fig 4A, bottom panel). These TP4 peptides at 0.25x, 0.5x or 1x Sar exhibited multiple bands anywhere from monomers, dimers to hexamers and more aggregates on SDS-PAGE after being crosslinked with 0.1% GA (10 mM). However, multiple bands were not seen at 2x or 4x Sar (Fig 4A top panel). The oligomerization status of TP4 peptides in 1x Sar was resistant to 300 mM NaCl or 30% trifluoroethanol (TFE), but susceptible to 40% TFE or more (Fig 4B and 4C).

### Anionic charges on the surface of TP4 assembly

**Fig 5:**
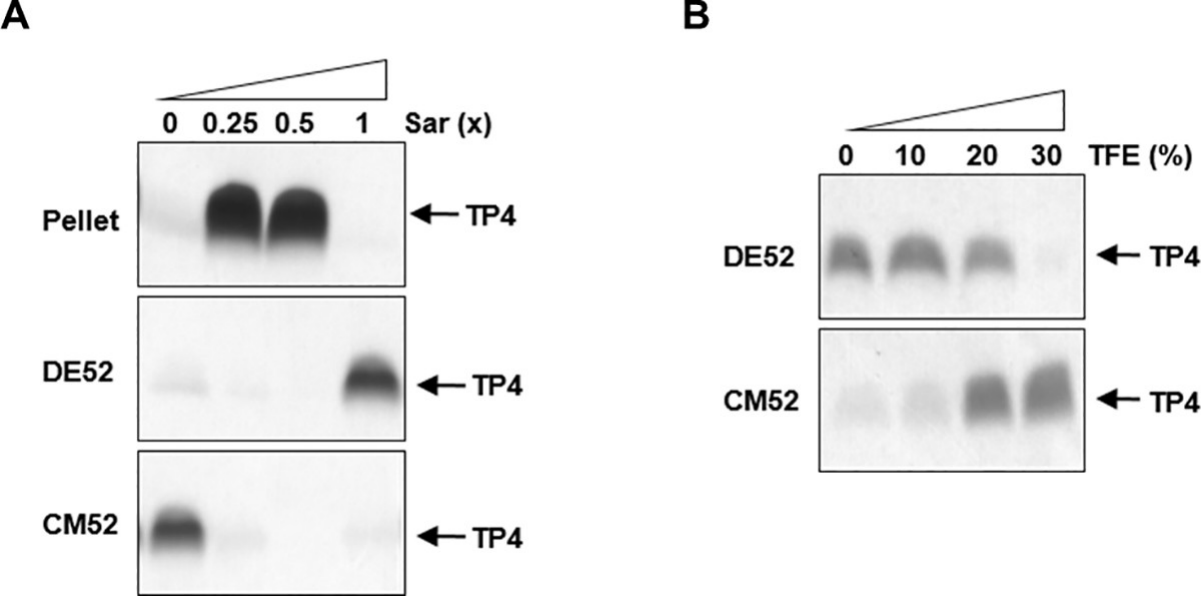
Anionic charges on the surface of TP4 oligomers. (A) Binding of TP4 peptides to cationic (DE52) and anionic (CM52) resins. TP4 peptides were dissolved in sarkosyl solutions and centrifuged. The soluble fractions were incubated with DE52 or CM52 resins. The TP4 peptides in 0x Sar were pulled down by CM52 resins and those in 1x Sar were pulled down by DE52 resins. (B) Anionic charges on the surface of TP4 oligomer were altered by TFE. Various concentrations of TFE were added to TP4 peptides which are soluble in 1x Sar, and incubated with DE52 or CM52 resins. 30% TFE enabled anionic TP4 oligomers to bind CM52 resins at 1x Sar, but became unable to bind DE52 resins.

Similar to the results shown in Fig 4A, the TP4 peptides were soluble in 1x or 0x Sar (without sarkosyl) while they precipitated at 0.25x or 0.5x Sar (Fig 5A). In the absence of sarkosyl, the TP4 peptides (as monomer) were pulled down by anionic resins (carboxylmethyl-cellulose, CM52) but not by cationic resins (diethylaminoethyl-cellulose, DE52) due probably to the abundance of cationic arginine residues. In contrast, the TP4 peptides at 1x Sar (as oligomer) were pulled down by DE-52 resins, but not by CM-52 resins (Fig 5A). Interestingly, the TP4 peptides at 1x Sar became able to bind CM-52 resins but not DE-52 resins if TFE concentration was adjusted to 20–30% (v/v) (Fig 5B) at which the TP4 peptides still existed in oligomers as shown in Fig 4C. These results indicate that TP4 oligomers remain soluble at 1x Sar probably due to charge repulsion among TP4 assemblies having anionic sarkosyl on the surface.

### Interaction between different AMPs

**Fig 6:**
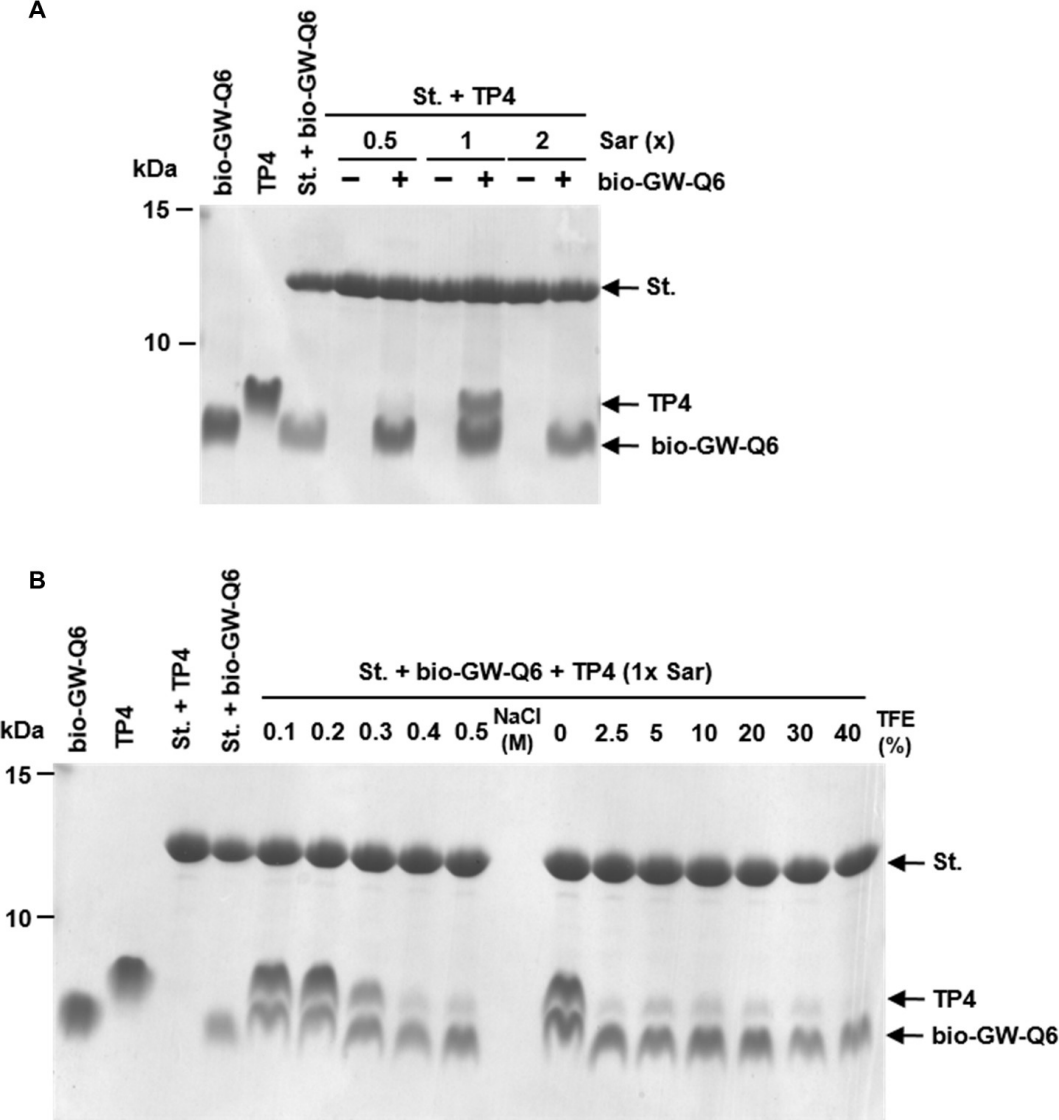
Hetero-binding of AMPs and susceptibility to salts and trifluoroethanol. (A) Pull down of TP4 assembly by biotinylated AMP GW-Q6. The soluble TP4 peptides (4 μg in 600 μl sarkosyl solution) were incubated with 6 μg biotinylated GW-Q6 which had been immobilized on streptavidin beads. The TP4 peptides were recognized and bound by biotinylated GW-Q6 only at 1x Sar. (B) Susceptibility of AMP assembly to salts and trifluoroethanol. The TP4 peptides bound to above-mentioned gels were eluted by 0.3 M NaCl or 2.5% TFE. Bio-GW-Q6, St. and TFE represent biotinylated GW-Q6, streptavidin-conjugated beads and trifluoroethanol.

Similar to the interactions between TP4 peptides, the TP4 assembly was able to bind other biotinylated AMPs such as GW-Q6 which are immobilized on the streptavidin gel at 1x Sar, but not at 0.5x or 2x Sar (Fig 6A). The hetero-binding between TP4 assembly and biotinylated GW-Q6 was resistant to 0.2 M NaCl and became labile at 0.3 M NaCl or higher (Fig 6B). However, these interactions were dramatically reduced by TFE as majority of the binding was abolished by 2.5% TFE (250 mM). Only small amounts of TP4 remained bound until 40% TFE (Fig 6B). These results indicate that hetero-binding between AMP and other AMP assemblies are still mainly mediated through hydrophobic interactions instead of electrostatic interaction.

### Morphology of TP4 assembly under different sarkosyl concentrations

**Fig 7:**
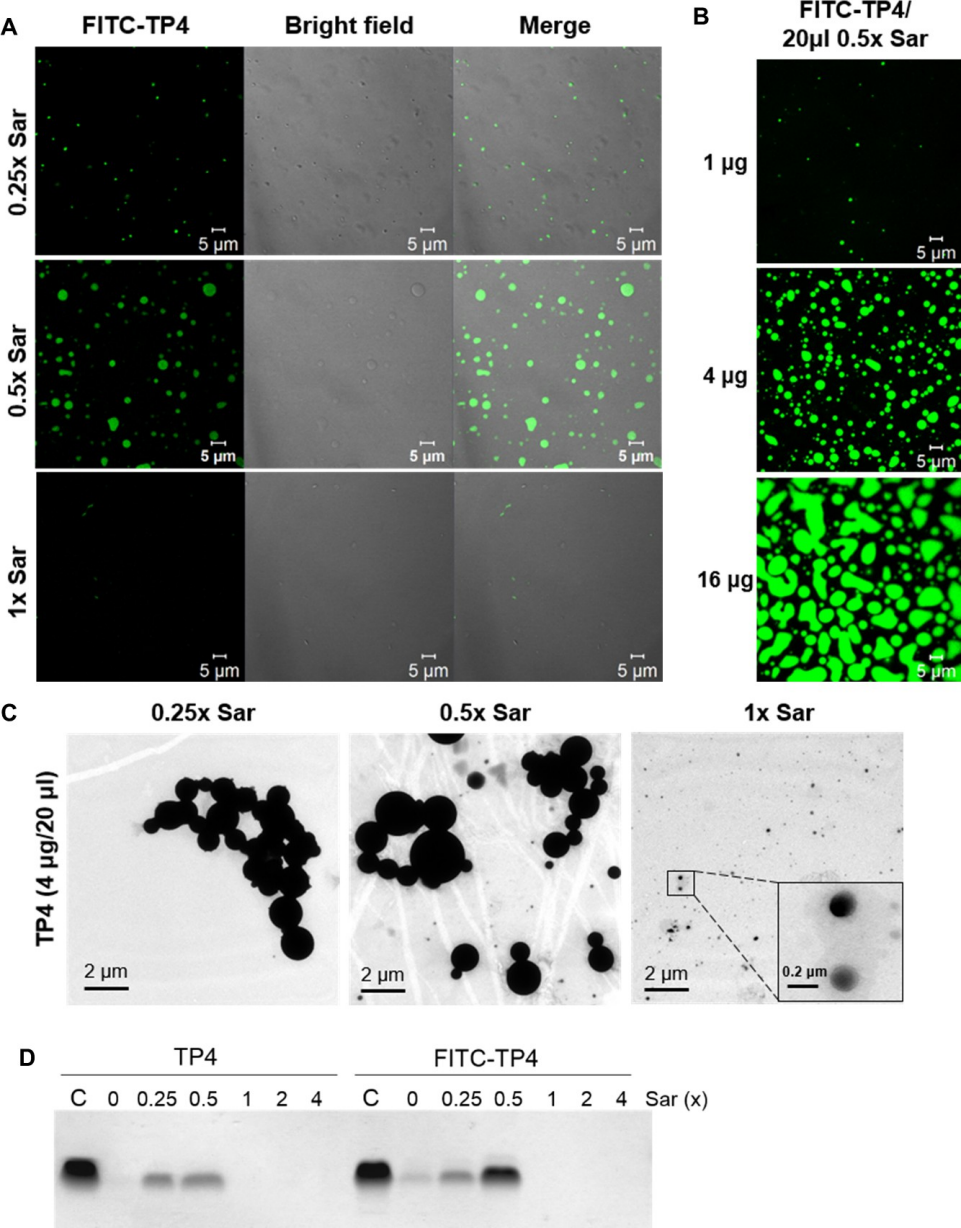
Morphologies of TP4 particles. (A) Morphology of FITC-TP4 particles in sarkosyl solutions. FITC-TP4 peptides (4 μg) were dissolved in 20 μl of 0.25x, 0.5x or 1x Sar solution and examined under fluorescence confocal microscope CLSM780. (B) Morphology of FITC-TP4 particles at different peptide concentrations. FITC-TP4 peptides were dissolved in 0.5x Sar (1, 4, 16 μg in 20 μl). The sizes of FITC-TP4 particles increased with the concentration of FITC-TP4 peptides. (C) Morphology of TP4 particles analyzed by transmittance electronic microscope. TP4 peptides (4 μg) were dissolved in 20 μl of 0.25x, 0.5x or 1x Sar solution and examined under transmittance electronic microscope JEM-1200EX. The sizes of TP4 particles at 0.25x and 0.5x Sar were larger than those at 1x Sar. (D) Precipitation of TP4 and FITC-TP4 peptides at various sarkosyl solutions. TP4 and FITC-TP4 (4 μg each) were dissolved in 600 μl sarkosyl solutions as indicated, and the precipitates were analyzed by SDS-PAGE after centrifugation at 13,200xg for 5 min.

Similar to TP4, the FITC-labelled TP4 solution was soluble and clear in appearance at 1x Sar, while they became opaque and insoluble at lower sarkosyl concentrations, especially at 0.5x (Fig 7D). To investigate the conformation of TP4 assembly, the morphologies of FITC-TP4 and TP4 peptides were examined under different sarkosyl concentrations by confocal fluorescence microscope (CFM) and transmittance electronic microscope (TEM). FITC-TP4 peptides appeared as small particles/vesicles with diameters of 0.2–5 μm at 0.5x Sar, but became smaller at 0.25x Sar and invisible at 1x Sar (Fig 7A). Despite being at the same sarkosyl concentration (0.5x Sar), the size of the particles increased with peptide concentration up to 20 μm in diameter (Fig 7B). The non-labelled TP4 particles also exhibited small particles with similar diameters of 0.2–2 μm at 0.25x and 0.5x Sar and became even smaller (0.1–0.2 μm) at 1x Sar when examined under TEM (Fig 7C). These results indicate that sarkosyl enables TP4 peptides to form oligomers in particles/vesicles.

### Formation of TP4 particles

**Fig 8:**
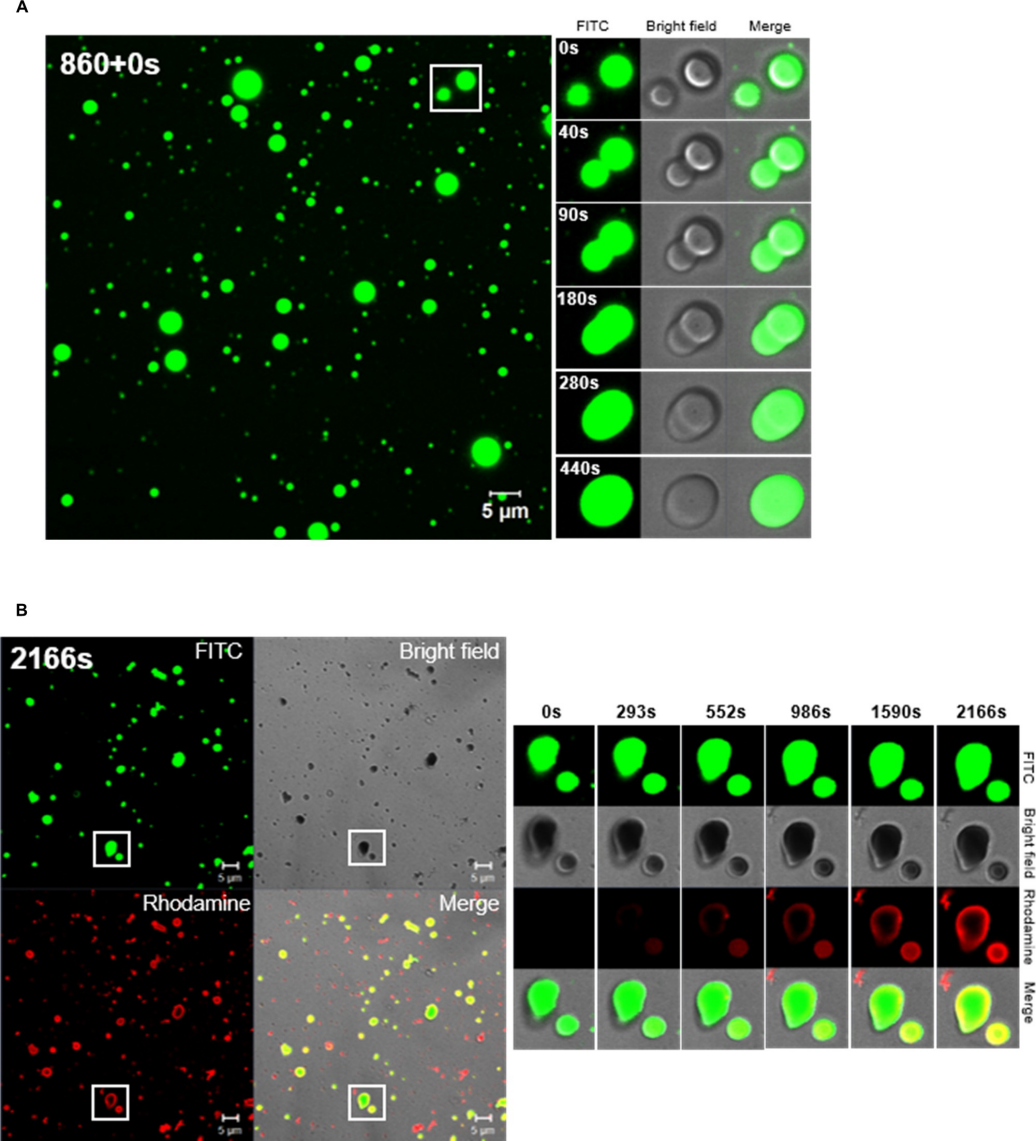
Formation of TP4 particles in the presence of sarkosyl. (A) Fusion of TP4 particles at 0.5x Sar in a time-dependent manner. FITC-TP4 peptides (4 μg) were dissolved in 20 μl 0.5x Sar, loaded on cover glass and examined by CLSM 780. TP4 peptides existed in small particles and fused with each other. The images generated in the processing of TP4 particle fusion was taken at 860s after dissolving TP4 peptides in 0.5x Sar (within the white frame of left panel), and were also shown on the right with three fields (fluorescence, bright field and merged image). (B) Deposition of Rhodamine-TP4 peptides on existing FITC-TP4 particles. Rhodamine-TP4 peptides (2 μg in 20 μl 0.5x Sar) were added to a drop containing preformed FITC-TP4 particles (2 μg in 20 μl 0.5x Sar) on cover glass and examined by CLSM780. The Rhodamine-TP4 peptides existed in small/red particles by themselves or deposited on surrounding FITC-TP4 particles. The images at the right panel showed Rhodamine-TP4 deposition on existing FITC-TP4 particles taken at 2166s after the addition of Rhodamine-TP4 peptides at 0.5x Sar within the white frame of the left panel. The FITC-TP4, Rhodamine-TP4 and FITC-TP4/Rhodamine-TP4 complex are shown in green, red and yellow.

At 0.5x Sar concentration, the green fluorescence FITC-TP4 peptides aggregated into small particles, fused together to form larger particles, and attached onto glass plate in a time-dependent manner (Fig 8A). Alternatively, exogenous Rhodamine-labelled TP4 peptides deposited on the existing green FITC-TP4 particles as shown in a yellow ring on the green particles on the glass. In addition, they may form particles in red by themselves (Fig 8B). Two major classes of large FITC-TP4 particles (solid and concave) as well as small particles were seen at 0.5x Sar. The three dimensional structures of solid particles (S1A Fig) and concave particles (S1B Fig) were shown. The fluorescence/phase-merged and fluorescence images were shown in panels a and b. The cross section images of solid FITC-TP4 particle were shown from bottom to top in the order of panel c to l and all were shown to be full (S1A Fig). It is worthy to note that some small particles were deposited on the top of large particles. In contrast, shadows were seen in some cross-sectional images of the concave particles (S1B Fig).

### Morphological change of TP4 particles caused by sarkosyl/TFE

**Fig 9:**
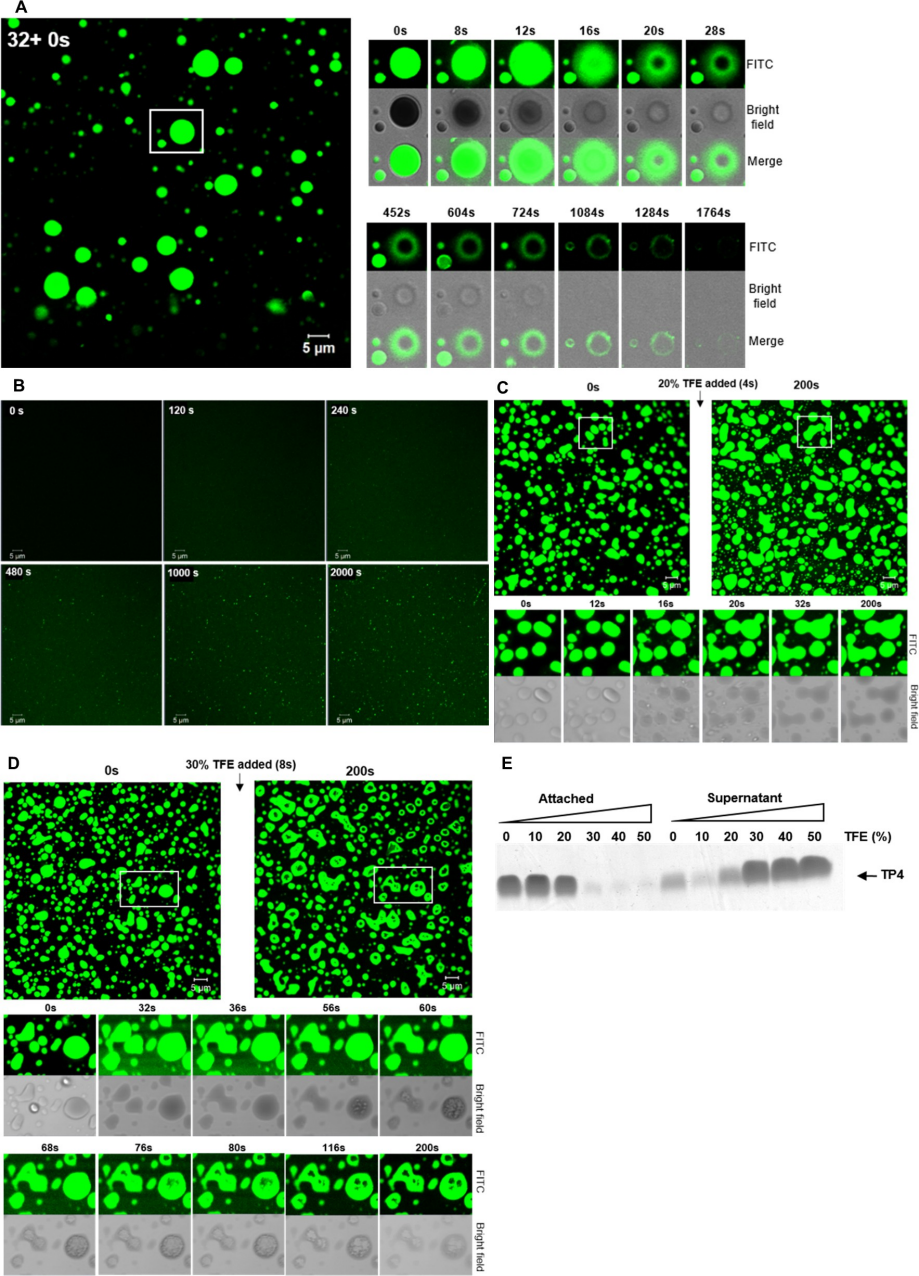
Conformational change of TP4 particles by sarkosyl and trifluoroethanol. (A) Disruption of FITC-TP4 particles by sarkosyl. 1x Sar was prepared by the addition of equal volumes of 1.5x Sar (20 μl) to FITC-TP4 particles-containing drop (4 μg in 20 μl 0.5x Sar) on cover glass. Images were taken a by CLSM780 in a time course as demonstrated in the right panel with three fields (fluorescence, bright field, and merged image). (B) Formation of FITC-TP4 vesicles in 0.5x Sar. FITC-TP4 peptides (3 μg) were dissolved in 20 μl 1x Sar and loaded on glass. Equal volumes of 0x Sar was added to the drop and images were taken and shown as mentioned above. (C, D) Fusion and disruption of FITC-TP4 particles by TFE. FITC-TP4 particles (4 μg in 20 μl 0.5x Sar) were prepared as mentioned in panel (A). The 0.5x Sar solution was drained off and replaced with 20% TFE and 30% TFE. The FITC-TP4 particles fused with each other in 20% TFE (C) and dissolved in 30% TFE (D). (E) Susceptibility of TP4 vesicles to 30% TFE. The preformed TP4 particles (8 μg/75 μl 0.5x Sar) were resuspended in 100 μl TFE solution for 1hr with gentle shaking. The TP4 peptides in the supernatants and pellets after centrifugation were analyzed by SDS-PAGE and Coomassie Blue staining.

To investigate the action of AMP on bacterial membrane, morphological changes of TP4 particles caused by either surfactant (sarkosyl) or hydrophobic solvent (TFE) were studied in a time-dependent manner. TP4 particles enlarged immediately (8 to 20 seconds) after the increase in concentration of sarkosyl from 0.5x to 1x Sar, then dissolved into solution from both internal and external sides of the particles and became invisible in 20–30 minutes (Fig 9A). In contrast, small TP4 particles re-appeared from the TP4-containing solution if 1x Sar was diluted to 0.5x Sar (Fig 9B). In addition, the preformed TP4 particles enlarged and fused together in less than one minute in 20% TFE (Fig 9C). However, these TP4 particles enlarged and dissolved in 30% TFE within three minutes with shadows inside the particles (Fig 9D). Alternatively, the non-labelled TP4 peptides which were deposited on the plastic wall of the Eppendorf tube at 0.5x Sar probably through hydrophobic interaction were resistant to 0–20% TFE, but were susceptible to 30–50% TFE (Fig 9E).

### Oligomerization of TP4 peptides on bacterial membrane

**Fig 10:**
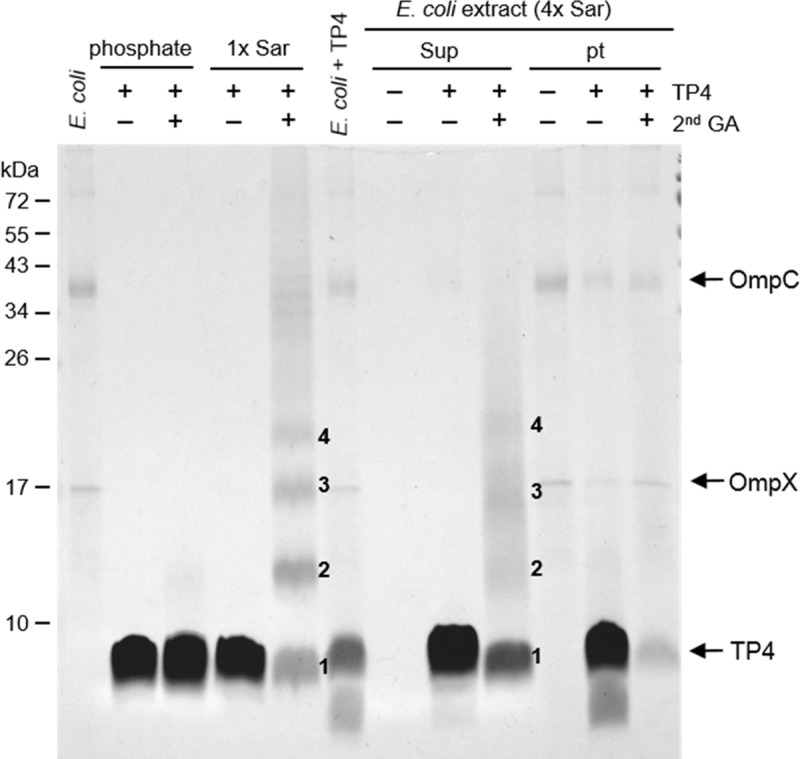
Assembly of TP4 peptides on the membrane of glutaraldehyde-fixed *E*. *coli*. The 0.2% glutaraldehyde-fixed *E*. *coli* cells were treated with 40 μg TP4 peptides, crosslinked by 0.1% glutaraldehyde, extracted by 4x Sar and analyzed by SDS-PAGE. The bound TP4 peptides from the 4x Sar extract exhibited multiple bands (1–4) on the gel, which are similar to those induced by 1x Sar, but those from the insoluble pellet did not. Numbers 1, 2, 3 and 4 on the gel represent monomers, dimers, trimers and tetramers, respectively. Phosphate, 10 mM sodium phosphate pH 7.4; Sup, supernatant; 2nd GA, 0.1% glutaraldehyde; OmpC, outer membrane protein C; OmpX, outer membrane protein X.

More than half of the bound TP4 were extracted from GA-fixed bacteria by 4x Sar which would not normally extract endogenous proteins from the bacteria. The TP4 peptides exhibited a ladder pattern on the SDS-PAGE similar to that of sarkosyl-induced TP4 oligomerization if the TP4-treated bacteria were cross-linked with 0.1% GA and extracted by 4x Sar (Fig 10). In contrast, no ladder was seen in the pellet. The outer membrane proteins OmpX and OmpC were extracted from GA-fixed *E*. *coli* by the heated SDS-PAGE loading buffer, but not by 4x Sar. These results suggest that TP4 peptides assemble into oligomers on/in the membrane through hydrophobic interaction before entering the bacteria.

## Discussion

Although thousands of AMPs have been isolated and investigated for several decades, some of them even under phase II/III clinical trials for the prevention and treatment of microbial infection, the mechanism of action is still not clearly understood. Several models, including the carpet model (detergent-like), barrel-stave and toroidal models have been proposed for the function of AMP in disrupting membrane integrity [7, 16, 17, 26, 27]. In the carpet model, peptides bind to the surface of cell membrane and render them into micellar structures just like detergents. In the barrel-stave model, peptides bind to the membrane surface and undergo a conformational change to adopt an amphipathic structure. They self-assemble and insert more deeply into the membrane forming a ring-like “barrel” pore. The toroidal mechanism is similar to the “barrel-stave” mode of pore formation. This differs from the barrel-stave pore as the peptides still interact with the lipid head groups but are not situated within the membrane hydrophobic core. This arrangement is not as stable as a barrel stave pore and is therefore more transient.

Although the proposed mechanisms are different, these AMPs have to be attached on the membrane before creating a hole or altering the membrane integrity. Our previous studies show that the α-helical as well as the amphipathic nature of the TP4 peptide can be driven *in vitro* by surfactants (sarkosyl or SDS), dodecylphosphocholine (DPC), trifluoroethanol (TFE) or lipopolysaccharides (LPS) [19, 20, 28]. In this report, we found that TP4 peptides are able to bind *E*. *coli* in just 3–5 minutes after addition which is in agreement with the sharp increase of permeability to SYTOX Green. The binding capacity of glutaraldehyde (GA)-fixed cells was close to one third of live cells. This indicates that TP4 peptides are internalized into the cytosol of live cells after binding. Interestingly, the TP4 peptides bound to GA-fixed bacteria could be extracted by sarkosyl (0.3%, 4x Sar) but were resistant to 0.3 M NaCl. Furthermore, they are shown to form oligomers on/in the bacterial membrane. These results suggest that binding or insertion of TP4 oligomers into the bacterial membrane is mainly mediated through hydrophobic interaction instead of electrostatic interaction.

To study the interaction of TP4 peptides with bacterial membrane, the anionic surfactant sarkosyl was employed to mimic the membrane which contains hydrophobic lipid acids and anionic residues. Sarkosyl is composed of a 12-carbon hydrophobic tail and an anionic carboxylate head-group tethered by an amide group [29]. It is similar to the components of membrane which also contains hydrophobic lipid acids and anionic residues. It has been shown to induce α–helical and amphipathic structures in TP4 peptides by 0.075% sarkosyl (2.6 mM), at which AMPs could bind to the receptor OprI/Lpp on the outer membrane of the Gram-negative bacteria [19, 20]. The conformational changes of TP4 peptides are reversible at the tested sarkosyl concentrations. The helix formation of TP4 peptide is also induced by similar surfactants such SDS and DPC (dodecylphosphocholine) [19]. Here we find that sarkosyl was able to drive TP4 peptides into oligomers either in soluble form (1x Sar or higher) or insoluble form (0.5x Sar or less). These interactions among TP4 components were resistant to 0.3 M NaCl or 30% TFE (Fig 4). The pull down experiment with cationic DE-52 resins suggests that soluble TP4 oligomers are coated with negatively charged sarkosyl on the surface (Fig 5). However, the insoluble oligomers are fused together and attach onto plastic/glass probably through hydrophobic interactions.

Regarding the stoichiometry of TP4 and sarkosyl in the TP4 complex, the ratio of TP4 to sarkosyl was not constant in the formation of TP4 particle. It is known that the molecular masses of TP4 and sarkosyl are 2,981 Da and 293 Da, respectively. The ratios of TP4 and sarkosyl in TP4 vesicles (4 μg in 20 μl 0.25x Sar) as shown in Fig 7A were 1:10 by molarity (65 μM TP4 to 640 μM sarkosyl), 7:10 by charge equilibrants (7 positive charges, 6R+1K, in TP4 and 1 negative charge in sarkosyl) and 1:1 by weight (4 μg TP4 to 3.75 μg sarkosyl). In addition, the size and solubility of TP4 particles varied with the TP4/sarkosyl ratio and concentration of TP4 employed as shown in Fig 7B. In contrast, TP4 peptides may dissolve into solution if the sarkosyl/TP4 ratio or sarkosyl concentration was increased as shown in Fig 7A and 7D.

The conformational change of the TP4 particle increased immediately after the increase of sarkosly concentration from 0.5x to 1x Sar and dissolved gradually into solution from both internal and external sides of the particle. In contrast, the TP4 particles re-appeared when the sarkosyl buffer was diluted from 1x to 0.5x Sar. Furthermore, the TP4 particles fused together or dissolved into solution depending on the concentrations of TFE employed. Therefore, this suggests that the TP4 and sarkosyl by themselves may assemble into layers through parallel hydrophobic interactions, then form a bilayer through head-to-head electrostatic interaction and leaving the hydrophobic face outward. As the concentration of sarkosyl increases, they may insert into sarkosyl layers, wrap the TP4 layer and break the TP4 large particle into smaller particles having TP4 layer inside and sarkosyl layer outside. The excess sarkosyl may attach to the hydrophobic face of the outer layer sarkosyl in a tail-to-tail manner through hydrophobic interaction leaving anionic charge outward. The attached sarkosyl molecules on the surface of TP4 particles may be removed or competed off by excess amounts of TFE.

**Fig 11:**
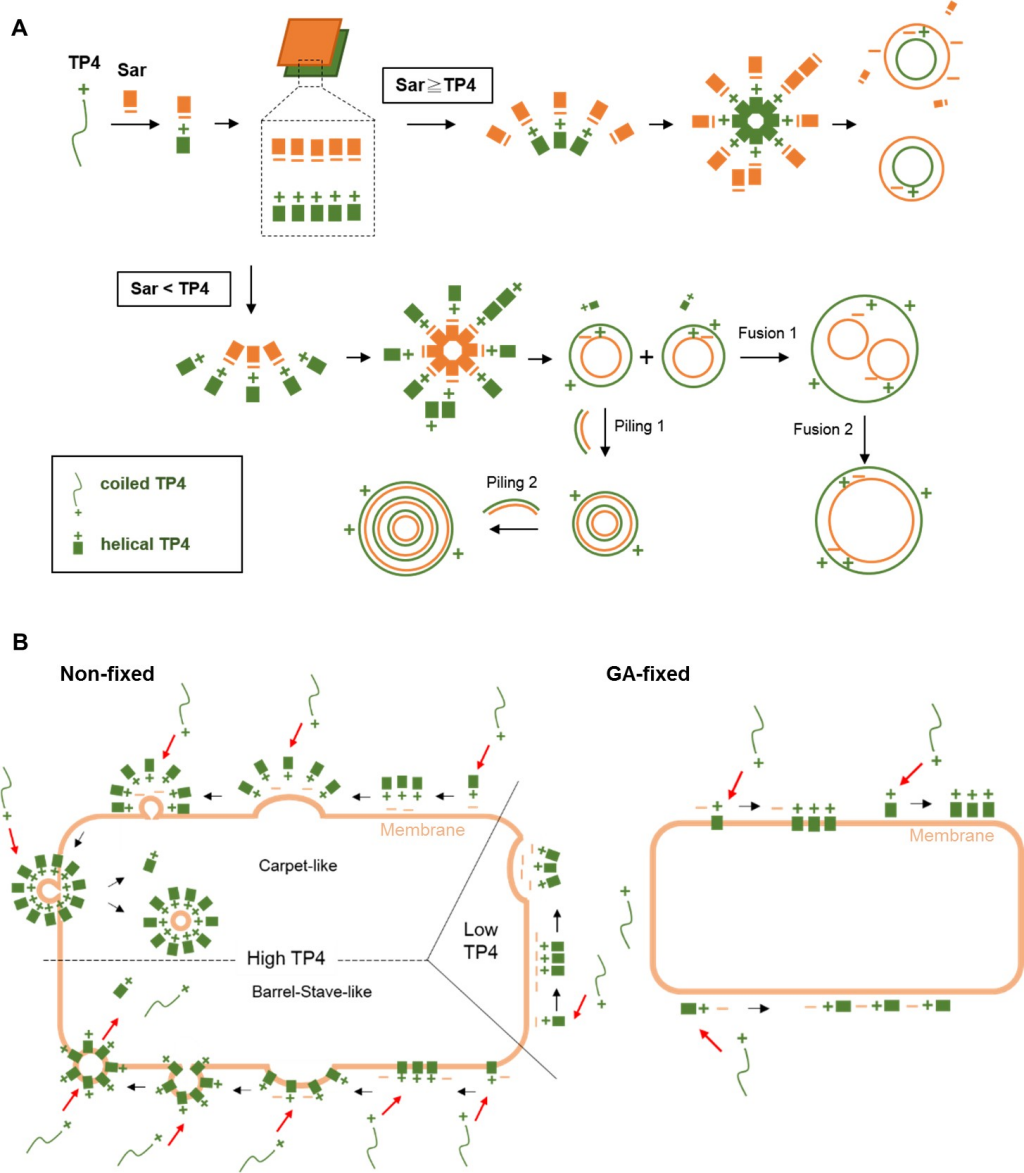
Proposed model of AMP assembly and entry into *E*. *coli*. (A). Assembly of cationic TP4 peptides into vesicles by sarkosyl (Sar). The TP4 peptides (green) are driven into α-helical and amphipathic structure by sarkosyl (orange). The TP4 peptides and sarkosyl are aligned into individual plane through hydrophobic interactions, then assembled into TP4/sarkosyl bilayers by electrostatic interaction. Various kinds of vesicle may be formed from these bilayers depending on the concentration of TP4 and sarkosyl employed. High ratios of sarkosyl/TP4 favor the formation of soluble and anionic vesicles, while lower ratios render the vesicles hydrophobic and insoluble in aqueous solution. In other words, high concentrations of TP4 peptides favor the fusion of small vesicles into large vesicles by hydrophobic interaction. (B) Entry of TP4 peptides into *E*. *coli*. In non-fixed cells, TP4 peptides are likely to cluster and form a bilayer with the bacterial membrane by electrostatic interaction, then form inward curvatures with bacterial membrane at low TP4 concentrations and form outward vesicles at high TP4 concentrations in a carpet-like mode. Alternatively, the hydrophobic core of clustered TP4 peptides may insert into the bacterial membrane in barrel-stave-like mode which resembles the enlargement of TP4/sarkosyl vesicles caused by sarkosyl or TFE addition. Since the formation of TP4/sarkosyl vesicle is reversible, TP4 peptides can be taken from medium and released into cytosol through the assembly and insertion on bacterial membrane. GA-fixed bacteria are not permeable to SYTOX Green and the binding of TP4 peptides to the bacteria membrane is suggested to be mediated through hydrophobic interaction.

The FITC conjugated TP4 peptides bind to *E*. *coli* within 3–5 minutes after peptide addition. The binding capacity of live cells was higher than that of glutaraldehyde-fixed cells which only allowed TP4 to bind on the membrane. The TP4 peptides formed oligomers on the membrane of *E*. *coli* when visualized by cross-linking and SDS-PAGE, and in small dots as observed by confocal fluorescent microscope. Based on the *in vivo* polymeric properties of TP4 peptides on bacteria membrane and *in vitro* dynamic properties of TP4 peptides including α-helix and particle formation, the model of TP4 assembly and subsequent actions on bacterial membrane are proposed and demonstrated in Fig 11. First, linear TP4 peptides are driven into α-helical and amphipathic structures by sarkosyl which resembles the membrane lipid environment containing a hydrophobic tail and an anionic group. Second, both TP4 peptide and sarkosyl themselves are aligned in an individual plane through parallel hydrophobic interactions, then form bilayers by head-to-head ionic interactions between the positively charged residues of TP4 and anionic group of sarkosyl, thereby leaving the hydrophobic faces outward. In bacteria, TP4 peptides are suggested to form clusters or a layer with anionic groups on membrane through electrostatic interaction, similar to lipopolysaccharide. Third, at low concentrations of TP4 or low TP4/sarkosyl ratio, the TP4/sarkosyl bilayers assemble only in small vesicles (TP4 inside/sarkosy outside) leaving excess sarkosyl outward. In bacteria, the low amounts of TP4 peptides are only able to form clusters or curvatures, but insufficient to form vesicles on the bacterial membrane. Fourth, if the TP4/sarkosyl ratio reaches a threshold, the TP4/sarkosyl bilayer may form seeding micelles (sarkosyl inside/TP4 outside) leaving excess TP4 outward. It is of note that the parallel hydrophobic interactions among TP4 molecules in TP4 layer may be stronger than those in sarkosyl layer because the mass and hydrophobic core of TP4 being larger than that of sarkosyl, 2943 Da and 293 Da, respectively. These TP4 particles have excess surface TP4 grow into larger vesicles by the deposition of TP4/sarkosyl bilayers or the fusion of existing small vesicles which possess hydrophobic surfaces. Therefore, the TP4 layers are more prone to fuse with each other than the sarkosyl layers. With respect to the behaviors of TP4 peptides on bacterial membrane, they are proposed to cluster and form bilayers or vesicles with membrane inside and TP4 layer outside known as Carpet-like mode. As mentioned above, the formation of TP4/sarkosyl particles is reversible depending on sarkosyl concentration, the exogenous TP4 peptides in solution may incorporate into the TP4 layer and then release into cytosol either in monomer or TP4/membrane vesicle. Alternatively, the TP4/sarkosyl vesicles were shown to enlarge immediately after the addition of sarkosyl (1x Sar) or TFE (30%) (Fig 9A and 9D). Thus the sarkosyl or TFE molecules are suggested to insert into the sarkosyl layer of TP4/sarkosyl vesicles and the exogenous TP4 peptides from the culture medium are proposed to cluster, insert into bacterial membrane and release into cytosol, called Barrel-stave-like model. Both live and GA-fixed bacteria were able to bind TP4 peptides with different capacity, but only live cells are permeable to SYTOX Green. This indicates that other membrane proteins of *E*. *coli* such like Lpp may be involved in the entry of TP4 peptides and increase of membrane permeability.

## Supporting information

S1 Table List of the most abundant proteins identified from the interested bands on SDS-PAGE. (DOCX)

S1 Fig Cross section of TP4 vesicle in 0.5x Sar. FITC-TP4 vesicles were shown in solid structure (A) and in concave structure (B) under fluorescence confocal microscope CLSM780. The selected FTIC-TP4 vesicles (6μg in 20μl 0.5x Sar) on cover glass with diameter larger than 10μm were selected for the analysis. The images were taken along Z-axis from bottom (cover glass side) to top by CLSM780. The images were assembled to form a three-dimensional model. Panel a and b shown in three-dimensional model with a cut-off plane along X-axis with merged image (fluorescence with bright field) and fluorescence image, respectively. Panel c to l (Figure A) and panel c to n (Figure B) showed all cross sections of TP4 vesicle from bottom to top.
(TIF)
