# Supplementary material for: Oligomerization and insertion of antimicrobial peptide TP4 on bacterial membrane and membrane-mimicking surfactant sarkosyl
Source: PLoS One. 2019 May 13;14(5):e0216946. doi: 10.1371/journal.pone.0216946 (PMC6513090; doi:10.1371/journal.pone.0216946)
Supplement: S1 Table — (DOCX) [file pone.0216946.s001.docx]

| **Band** | **Accession # of UniProKB** | **Protein** | **Score** | **# of Identified Peptides** | **Sequence Coverage** |
| --- | --- | --- | --- | --- | --- |
| Omp C | P06996 (OMPC_ECOLI) | Outer membrane protein X | 31288 | 31 | 73% |
| Omp X | P0A917 (OMPX_ECOLI) | Outer membrane protein C | 6388 | 11 | 53% |

The MS raw data were searched against the UniProtKB *Escherichia coli* database. The output revealed 31 peptides with 73% sequence coverage for outer membrane protein C in OmpC band and 11 peptides with 53% sequence coverage for outer membrane protein X in band Omp X.
