# Supplementary figures and images for: Oligomerization and insertion of antimicrobial peptide TP4 on bacterial membrane and membrane-mimicking surfactant sarkosyl
Source: PLoS One. 2019 May 13;14(5):e0216946. doi: 10.1371/journal.pone.0216946 (PMC6513090; doi:10.1371/journal.pone.0216946)

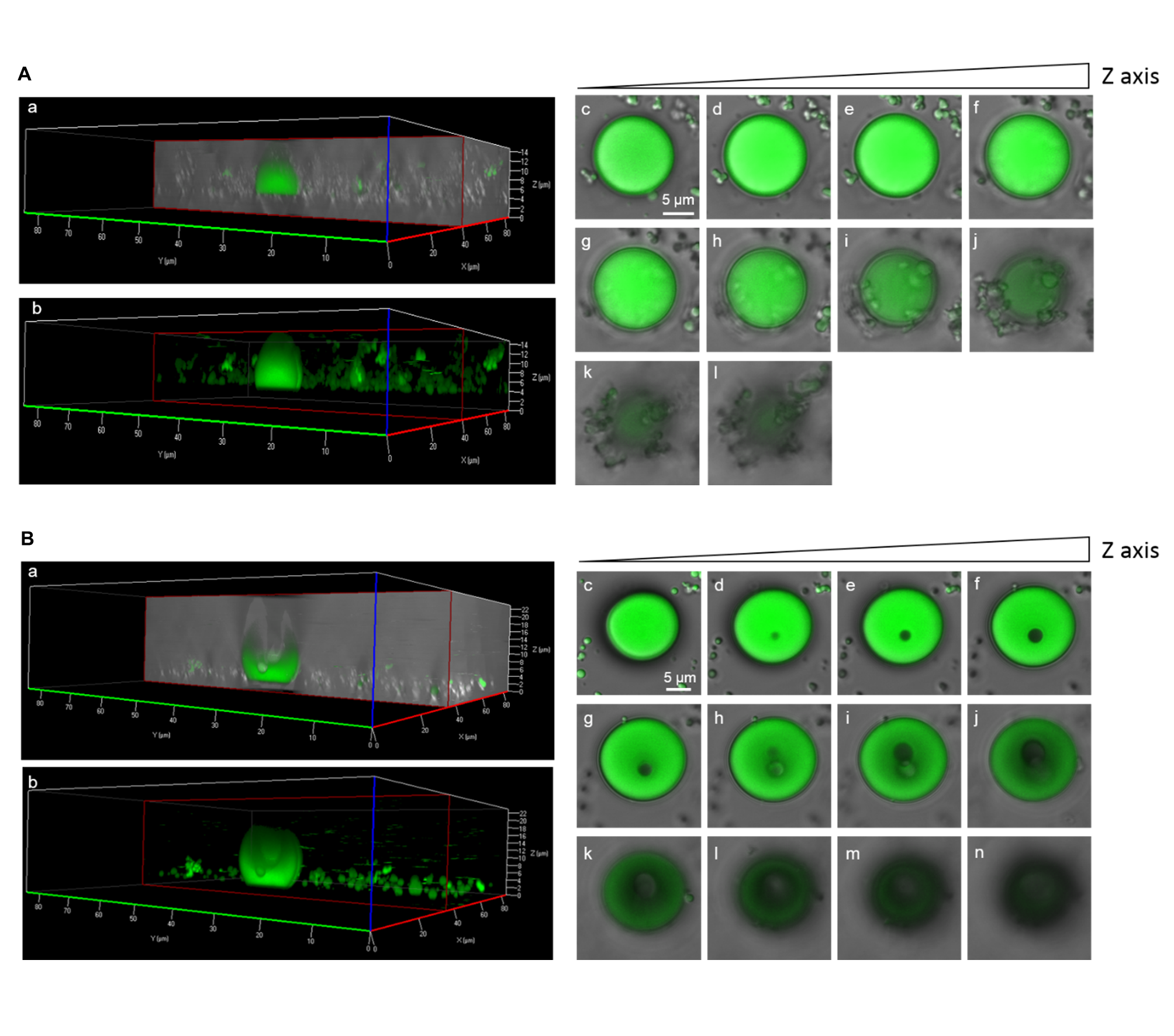

Supplement: S1 Fig — FITC-TP4 vesicles were shown in solid structure (A) and in concave structure (B) under fluorescence confocal microscope CLSM780. The selected FTIC-TP4 vesicles (6μg in 20μl 0.5x Sar) on cover glass with diameter larger than 10μm were selected for the analysis. The images were taken along Z-axis from bottom (cover glass side) to top by CLSM780. The images were assembled to form a three-dimensional model. Panel a and b shown in three-dimensional model with a cut-off plane along X-axis with merged image (fluorescence with bright field) and fluorescence image, respectively. Panel c to l (Figure A) and panel c to n (Figure B) showed all cross sections of TP4 vesicle from bottom to top. (TIF) [file pone.0216946.s002.tif]
